# Reliability and Reproducibility of Hadamard Encoded Pseudo-Continuous Arterial Spin Labeling in Healthy Elderly

**DOI:** 10.3389/fnins.2021.711898

**Published:** 2021-08-19

**Authors:** Katja Neumann, Martin Schidlowski, Matthias Günther, Tony Stöcker, Emrah Düzel

**Affiliations:** ^1^German Center for Neurodegenerative Diseases (DZNE), Magdeburg, Germany; ^2^German Center for Neurodegenerative Diseases (DZNE), Bonn, Germany; ^3^Department of Epileptology, University of Bonn Medical Center, Bonn, Germany; ^4^Fraunhofer Institute for Digital Medicine MEVIS, Bremen, Germany; ^5^MR-Imaging and Spectroscopy, University of Bremen, Bremen, Germany; ^6^Department for Physics and Astronomy, University of Bonn, Bonn, Germany; ^7^Institute of Cognitive Neurology and Dementia Research, Otto von Guericke University Magdeburg, Magdeburg, Germany; ^8^Institute of Cognitive Neuroscience, University College London, London, United Kingdom; ^9^Center for Behavioral Brain Science, Magdeburg, Germany

**Keywords:** arterial spin labeling, pCASL, reliability, reproducibility, perfusion, cerebral blood flow, arterial transit time, hadamard encoding

## Abstract

The perfusion parameters cerebral blood flow (CBF) and arterial transit time (ATT) measured with arterial spin labeling (ASL) magnetic resonance imaging (MRI) provide valuable essentials to assess the integrity of cerebral tissue. Brain perfusion changes, due to aging, an intervention, or neurodegenerative diseases for example, could be investigated in longitudinal ASL studies with reliable ASL sequences. Generally, pseudo-continuous ASL (pCASL) is preferred because of its larger signal-to-noise ratio (SNR) compared to pulsed ASL (PASL) techniques. Available pCASL versions differ regarding their feature details. To date only little is known about the reliability and reproducibility of CBF and ATT measures obtained with the innovative Hadamard encoded pCASL variant, especially if applied on participants in old age. Therefore, we investigated an in-house developed Hadamard encoded pCASL sequence on a group of healthy elderly at two different 3 Tesla Siemens MRI systems (Skyra and mMR Biograph) and evaluated CBF and ATT reliability and reproducibility for several regions-of-interests (ROI). Calculated within-subject coefficients of variation (wsCV) demonstrated an excellent reliability of perfusion measures, whereas ATT appeared to be even more reliable than CBF [e.g., wsCV(CBF) = 2.9% vs. wsCV(ATT) = 2.3% for a gray matter (GM) ROI on Skyra system]. Additionally, a substantial agreement of perfusion values acquired on both MRI systems with an inter-session interval of 78 ± 17.6 days was shown by high corresponding intra-class correlation (ICC) coefficients [e.g., ICC(CBF) = 0.704 and ICC(ATT) = 0.754 for a GM ROI]. The usability of this novel Hadamard encoded pCASL sequence might improve future follow-up perfusion studies of the aging and/or diseased brain.

## Introduction

Arterial Spin Labeling (ASL) is a magnetic resonance imaging (MRI) technique that provides insights to microvascular brain perfusion (Williams et al., 1992). One important parameter to characterize the cerebral health condition with ASL is the cerebral blood flow (CBF; Lassen, 1959). CBF gives the volume of blood [in unit (ml)] being delivered to a certain mass of tissue (usually 100 g is used) during a given time period (e.g., 1 min). This results in the common unit (ml/100 g/min) for CBF. Larger CBF values indicate that either “more” blood is passing the tissue within a certain time, or/and if the blood volume is assumed to be constant, that the blood passage itself is faster. Since a few years another perfusion parameter had become the focus of attention: the arterial transit time (ATT). The ATT of a region-of-interest (ROI) defines how long arterial blood needs to travel from the labeling region to that ROI. The spatial structure of the cerebral vasculature reflects the respective demand of oxygen and nutrients in gray and white matter tissue and its need to adapt appropriately to changing conditions. This explains why the ATT for gray matter is usually lower than for white matter. The importance of an ATT estimation to define cerebral health was confirmed in several studies. One example is the study published by Paling et al. (2014) of multiple sclerosis (MS) patients. They found a prolonged ATT of gray and white matter in MS patients compared to healthy controls, whereas higher ATTs were connected to the severity of disease specific disabilities. An accurate measure of the ATT is also valuable for a correct CBF quantification.

For perfusion imaging with ASL, images with prior arterial blood labeling (so-called tag images) are subtracted from those without labeling (control images) to calculate perfusion weighted images (Williams et al., 1992). There are different labeling strategies. In pseudo-continuous ASL (pCASL) blood water spins passing a thin imaging plane are continuously inverted by a series of short radiofrequency (RF) pulses and correspondingly pulsed gradient fields (Dai et al., 2008). Whereas in pulsed ASL (PASL) a single RF pulse is used to invert blood water spins of a relatively large slab (Wong et al., 1998). Generally, perfusion-weighted ASL images suffer from an intrinsically low signal-to-noise ratio (SNR) because the perfusion signal originates from the blood water spins, which yield only a relatively small portion compared to brain tissue. The use of larger voxels in ASL acquisitions compensates the low SNR to a certain degree, but necessitates the application of a proper partial volume correction (PVC) method along with the perfusion quantification process (Chappell et al., 2011). The PVC differentiates gray and white matter contributions of the cumulative perfusion signal of each voxel.

Arterial spin labeling is non-invasive and uses endogenous blood water instead of exogenous contrast agents. It is therefore suitable for follow-up studies (Weber et al., 2003). Especially in longitudinal multi-center studies good reliability and reproducibility of measurements is needed. Chen et al. (2011) demonstrated a higher test-retest reliability of pCASL compared to PASL scans in 2011. They also compared scans with an inter-session interval of 1 week and showed that in their study the reproducibility of pCASL and PASL was less different than the test-rest reliability (Chen et al., 2011). Furthermore, the use of pseudo-continuous labeling was widely recommended by leading ASL experts as published by Alsop et al. (2015). Two years later, Dolui et al. (2017) published their results of a comparison study of PASL and pCASL acquisitions on patients with mild cognitive impairment (MCI), where they reported background suppressed (BS) 3D pCASL to be more suitable to detect CBF changes in MCI patients.

Guenther (2007) introduced a time-efficient encoding approach for multi inversion time ASL, called Hadamard encoded or time-encoded ASL. In Hadamard encoded pCASL the bolus is divided into several subboli, each with a different post labeling delay (PLD). Nevertheless, there are only few studies investigating the reliability of 3D pCASL with Hadamard encoded multiple PLDs. While Cohen et al. (2020) evaluated such reproducibility on healthy young males using a 7-PLD sequence, Guo et al. (2018) differentiated the greater reproducibility of CBF values of a 5-PLD sequence compared to a better reproducibility of ATT values of a 7-PLD setting.

In our study we evaluated the reliability and reproducibility of CBF and ATT values obtained with a 7-block Hadamard encoded 3D pCASL sequence on healthy elderly.

## Materials and Methods

### Participants and MRI Acquisition

Twenty six healthy elderly participants [between 56 and 84 (68.3 ± 6.3) years old, 15 females] participated in this study approved by the Ethics Committee of the Otto von Guericke University Magdeburg, Germany. Signed consent was provided by each participant.

The participants underwent two MRI acquisitions with a mean inter-session interval of 78 ± 17.6 days. The first session was conducted at a 3 Tesla (T) Siemens Magnetom Skyra MRI scanner (Siemens, Erlangen, Germany) with a 32-channel head coil (Nova Medical, Wilmington, MA, United States). Two runs with identical protocol of a pCASL and one PASL scan with FAIR labeling and 3D-GRASE (Günther et al., 2005) readout were acquired. The in-house developed pCASL sequence (Boland et al., 2018) used an unbalanced Hadamard encoded labeling with VERSE RF pulses. Compensation of T1-relaxation related signal decay (van Osch et al., 2018) within the subbolus range was realized via a seven-block Hadamard scheme with 500 ms PLD and block durations of 224/257/305/374/482/683/1175 ms. This resulted in effective PLDs of 500/724/981/1286/1660/2142/2825 ms. Two background suppression pulses were optimized and calculated dependent on the total ASL preparation time available (label duration + PLD) to obtain a 10% residual longitudinal magnetization of GM, WM, and CSF contributions at the end of the PLD to minimize signal contributions from static tissue. Additional proton density weighted images with a repetition time (TR) of 6 s were acquired with both phase encoding polarities for a calibration of the estimated perfusion. Further acquisition parameters were: TR/TE = 7000/22.9 ms, CAIPIRINHA *R* = 2 × 2 (Δ = 1), 3.3 mm isotropic resolution, 211 mm × 211 mm field-of-view (FOV), 36 slices, single-shot acquisition with band width (BW) = 2367 Hz/Px, EPI factor 32, turbo factor (TF) 18, resulting in a total acquisition time (TA) of 7:12 min. A prolonged TR of 7 s was chosen to remain within SAR limits and to ensure an equal scan time in all participants. With fast phase-contrast-based vascular scout images, the pCASL labeling plane was positioned such that feeding arteries led perpendicular as possible through it to maximize labeling efficiency. The PASL sequence variant (Günther et al., 2005) used FOCI RF pulses for a FAIR labeling and Q2TIPS for the suppression of background signal contributions. The PASL scan had the following parameters: TR/TE = 3800/22.32 ms, 4 mm × 4 mm × 5.5 mm voxel size, 256 mm × 192 mm FOV, 16 slices, BW = 2298 Hz/Px, EPI factor 32, TF = 12, two segments, two averaged multi-inversion time (TI) measurements with 13 TIs starting from 300 ms with an increment of 200 ms, BL = 1400 ms, 5 calibration scans with TR = 4360 ms and a TA of 7:08 min. The proton density weighted calibration scans were used to quantify perfusion weighted images.

Two additional pCASL scans (with identical parameters as mentioned above) were acquired during a second session at a 3T Siemens Biograph mMR (PET/MR) scanner using 32-channel head coil. Furthermore, a structural whole-brain T1-weighted MPRAGE sequence (Mugler, and Brookeman, 1990) with 1 mm isotropic resolution and TR/TE/TI = 2500/3.47/1100 ms was measured at each session.

### Structural Pre-processing and Region-of-Interest Definition

All structural T1-weighted scans were pre-processed using tools of the Oxford Centre for functional MRI of the BRAIN (FMRIB) Software Library (FSL) (Jenkinson et al., 2012). This step included a tissue-type segmentation [with FAST tool (Zhang et al., 2001)] and a subcortical structure segmentation [with FIRST tool (Patenaude et al., 2011)], which was used as a technical base to perform a PVC (Chappell et al., 2011) of perfusion images. Those segmentations were further used to define bilateral ROIs of the hippocampus, amygdala, caudate and accumbens. Tissue-type density maps were thresholded with a value of 0.9 and subsequently binarized to generate gray and white matter masks of each participant.

### Arterial Spin Labeling Perfusion Analysis

Voxel-wise estimation of CBF and ATT was performed using FSL’s BASIL toolkit (Chappell et al., 2009, 2010; Groves et al., 2009). The computation of perfusion parameters with BASIL was based on the general kinetic model (Buxton et al., 1998) and a variational Bayes approach (Chappell et al., 2010).

The analysis included a linear registration of individual ASL control and tag images as well as calibration images prior to image subtraction (Jenkinson et al., 2002). The resulting six individual motion parameters (three for rotation and three for translation) from this rigid body registration were later additionally used to compare pCASL and PASL acquisitions and to identify possible outliers. The Hadamard encoded pCASL data were analyzed analogously to the multi-PLD data, except that a decoding was performed instead of a pairwise control-tag subtraction and thereby perfusion-weighted images of the encoded PLDs were obtained. The calibration or proton density weighted scans represent the equilibrium magnetization and serve as a reference to convert quantified perfusion values from arbitrary to physiological values. The first scan of the obtained calibration scans was removed to ensure steady state signal. The remaining calibration images are two pairs of acquisitions with anterior-posterior (AP) and posterior-anterior (PA) phase polarity. These reference scans (M0 images) were averaged per polarity and then used for a distortion correction (Smith et al., 2004). The obtained correction was then also applied to the ASL data. Furthermore, a PVC by means of deployed tissue-type density maps (Chappell et al., 2011) was part of the perfusion analysis.

In PASL acquisitions the label plane is much closer to the readout than in pCASL acquisitions. Therefore, labeled blood water reaches the tissue (e.g., gray matter) faster and the related ATTs are lower compared to pCASL. This is reflected in the kinetic modeling, as the ATT is a variable modeling parameter with the prior GM assumption of 1.3 s for pCASL and 1.0 s for PASL. Besides this, the ATT modeling of the two approaches differed in the number and range of PLDs available.

### Statistical Analysis

The test-retest reliability was assessed by calculating the within-subject coefficient of variation (wsCV) as follows: $\text{wsCV}=\sqrt{\text{Mean}\,\left(\frac{\mathrm{wsVar}}{{\mathrm{wsMean}}^{2}}\right)}$, where wsVar is the within-subject variation and wsMean the mean value for both runs.

IBM SPSS Statistics 23 was used to obtain two-way mixed intra-class correlation (ICC; Koo and Li, 2016) coefficients to evaluate the inter-session reproducibility of pCASL scans and the intra-session reproducibility by comparing pCASL and PASL. Furthermore, mean ROI-based CBF and ATT values were tested for normality using Shapiro–Wilk test. SPSS was also used to perform analysis of the variance (ANOVA) for group comparisons, and for a general linear model analysis on the perfusion results.

A relation of ATT to the CBF estimation was evaluated by calculating Pearson’s correlation coefficients of corresponding ATT and CBF values.

## Results

### Quality Evaluation of ASL Scans

#### Motion

The head motion during the pCASL sequence on both MRI systems of all participants remained in an acceptable range (<2 mm), so that no participant had to be excluded from further analysis because of severe motion artifacts. Subsequently, pCASL acquisitions from the Skyra and the mMR Biograph were compared. An ANOVA showed that the overall translation in y-direction during the first run of the pCASL scan was significantly less if acquired at the Skyra system (mean y-translation at Skyra: 0.33 ± 0.13 mm; mean y-translation at mMR Biograph: 0.49 ± 0.22 mm; *p* = 0.005).

#### Signal-to-Noise Ratio

The temporal SNR (tSNR) within gray matter was computed by dividing the mean gray matter difference signal of all repetitions by its standard deviation and averaging the results of all PLDs. The results for the mean tSNR of the pCASL sequences from Skyra and mMR Biograph were comparable (for the Skyra tSNR = 1.72 ± 0.58 and for the mMR Biograph tSNR = 1.77 ± 0.36 for the mean of both runs) although we found significant differences of participants’ motion between both systems (see above).

#### Bolus Transition Through Gray Matter

**FIGURE 1:**
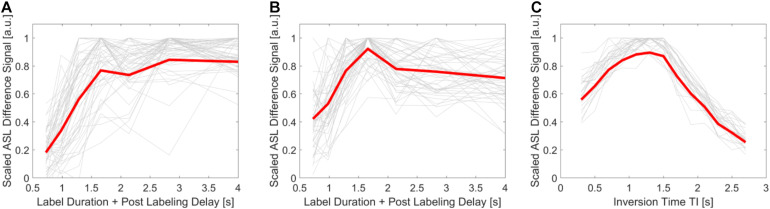
Visualization of the arterial spin labeling (ASL) bolus passage through the gray matter region-of-interest: The mean ASL difference signal at each measured or decoded time point is plotted separately for every participant. The mean of all lines is shown with a bold red line. In pseudo-continuous ASL (pCASL) acquisitions at the Skyra **(A)** and mMR Biograph **(B)** a compensation of the T1 related signal decay was applied. This is shown by an almost horizontal line of the mean ASL difference signal at later time points, opposite to the signal decrease in pulsed ASL (PASL) acquisitions at the Skyra **(C)**, where this method was not used. The mean ASL difference signal was scaled by dividing all values of each individual curve by its maximum.

The mean ASL difference signal at each inversion time was plotted for every participant (one line per participant; both pCASL runs at each system separately), see Figure 1. The bold red line represents the mean difference signal for all participants at each PLD. The T1 related signal decay could be successfully compensated in the pCASL acquisitions with the used Hadamard scheme, conveyed by a slope close to zero for later PLDs [(A), (B)]. It was not investigated whether this bolus shape also affects the accuracy of ATT estimations. The curve for the mean ASL difference signal for the PASL scan without a compensation for a T1 related signal decay shows a distinct bolus width and a greater signal decrease for later TIs (C).

### General Perfusion Results

**FIGURE 2:**
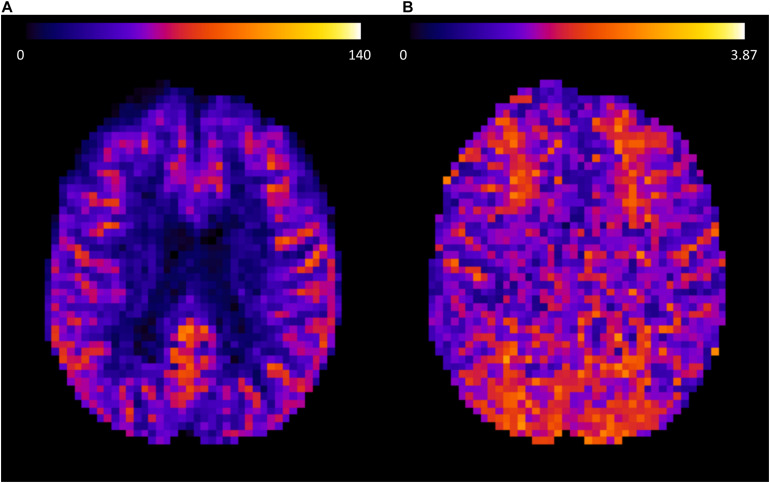
Axial slice of the cerebral blood flow (CBF) **(A)** and the arterial transit time (ATT) **(B)** result of a representative participant obtained from a pCASL acquisition at the Skyra MRI system. To visualize gray and white matter values within the same image, estimated results before a partial volume correction was applied are shown.

Table 1 lists mean results of CBF and ATT of various ROIs for the PASL scan and the two runs of the pCASL scan, both from Skyra and mMR Biograph acquisitions. Figure 2 shows an axial slice of the CBF and ATT result of the first run from a representative participant measured at the Skyra.

**TABLE 1:** Mean cerebral blood flow (CBF) and arterial transit time (ATT) for several region-of-interests (ROI) for pseudo-continuous ASL (pCASL) and pulsed ASL (PASL) acquisitions at 3T Siemens magnetic resonance imaging (MRI) systems (Skyra and mMR Biograph).

ROI	Perfusion parameter: CBF ± SD in [ml/100 g/min], ATT ± SD in [s]	Skyra			mMR Biograph	
			
		pCASL run 1	pCASL run 2	PASL	pCASL run 1	pCASL run 2
Gray matter	CBF	69.8 ± 12.7	68.6 ± 12.7	69.2 ± 10.6	65.3 ± 11.2	65.4 ± 11.8
	ATT	1.4 ± 0.13	1.4 ± 0.13	0.7 ± 0.08	1.3 ± 0.11	1.3 ± .10
White matter	CBF	28.2 ± 6.7	27.2 ± 7.0	41.7 ± 7.9	26.8 ± 5.1	27.2 ± 6.0
	ATT	1.5 ± 0.08	1.5 ± 0.10	1.0 ± 0.09	1.5 ± 0.07	1.5 ± 0.07
Left hippocampus	CBF	57.3 ± 10.1	57.3 ± 11.1	71.0 ± 13.0	53.1 ± 9.7	52.2 ± 12.5
	ATT	1.1 ± 0.14	1.2 ± 0.15	0.5 ± 0.06	1.1 ± 0.13	1.1 ± 0.10
Right hippocampus	CBF	57.3 ± 10.2	55.9 ± 10.9	73.1 ± 13.7	53.7 ± 9.3	52.5 ± 11.0
	ATT	1.2 ± 0.15	1.2 ± 0.16	0.5 ± 0.08	1.1 ± 0.12	1.1 ± 0.12
Left amygdala	CBF	47.0 ± 7.7	46.0 ± 8.7	51.7 ± 9.8	42.0 ± 7.6	41.9 ± 8.2
	ATT	1.1 ± 0.13	1.1 ± 0.12	0.5 ± 0.07	1.1 ± 0.12	1.1 ± 0.09
Right amygdala	CBF	47.0 ± 8.8	44.8 ± 8.9	55.3 ± 12.3	43.4 ± 6.5	42.7 ± 6.6
	ATT	1.1 ± 0.12	1.2 ± 0.13	0.5 ± 0.10	1.1 ± 0.13	1.1 ± 0.11
Left caudate	CBF	55.2 ± 11.4	54.5 ± 11.8	45.3 ± 10.4	55.7 ± 11.1	56.4 ± 11.0
	ATT	1.1 ± 0.14	1.1 ± 0.18	0.6 ± 0.11	1.1 ± 0.14	1.1 ± 0.12
Right caudate	CBF	52.7 ± 12.3	51.9 ± 12.3	52.0 ± 12.1	53.6 ± 10.0	53.8 ± 9.8
	ATT	1.1 ± 0.16	1.2 ± 0.16	0.5 ± 0.10	1.1 ± 0.14	1.1 ± 0.12
Left accumbens	CBF	73.3 ± 18.4	72.3 ± 18.6	54.2 ± 14.3	73.8 ± 14.0	71.1 ± 20.0
	ATT	1.0 ± 0.17	1.0 ± 0.20	0.8 ± 0.26	1.0 ± 0.16	1.0 ± 0.16
Right accumbens	CBF	61.2 ± 16.8	59.7 ± 14.1	63.9 ± 20.4	61.2 ± 8.8	58.9 ± 11.1
	ATT	1.1 ± 0.21	1.1 ± 0.21	0.5 ± 0.17	1.0 ± 0.17	1.0 ± 0.15

*The pCASL sequence was applied twice within the same session (results refer to run 1 and run 2).*

Both pCASL runs at each system led to comparable results for ATT and CBF, respectively. A one-way ANOVA confirmed that there were no significant differences for perfusion values of the first and the second pCASL run (*p* > 0.05).

However, with a general linear model analysis including the factors scanner, age and gender we found that the mean ATT was larger at Skyra acquisitions than those from the mMR Biograph for gray matter (*p* = 0.014), white matter (*p* = 0.022), left hippocampus (*p* = 0.025) and right hippocampus (*p* = 0.002). For all the other ROIs there were no differences detectable (*p* > 0.05). Thus, the factor scanner had also no effect (*p* = 0.189) on the mean gray-to-white matter contrast of CBF as shown via GLM analysis with age and gender as additional factors (Skyra: **GM_CBF_**/**WM_CBF_** = **2.52** ± ** 0.25**; mMR Biograph: **GM_CBF_**/**WM_CBF_** = **2.44** ± ** 0.17**). Altogether, the pCASL gray-to-white matter contrasts are higher than those for PASL (**GM_CBF_**/**WM_CBF_** = **1.68** ± ** 0.17**) and also higher than reported elsewhere, considering different pCASL sequences and different characteristics of the underlying cohort in those studies (Dolui et al., 2017; Vidorreta et al., 2017).

Additionally, we wanted to evaluate the time shift of the bolus arrival to gray and white matter and therefore computed the mean difference of the ATTs (△_**ATT**_ = **ATT_WM_** − **ATT_GM_**). At pCASL acquisitions from the Skyra the labeled bolus arrived about △_**ATT**_ = **0.13** ± **0.05s** later to white matter than to gray matter voxels. For pCASL acquisitions from the mMR Biograph the difference is △_**ATT**_ = **0.14** ± **0.04s** and for the PASL scan it is △_**ATT**_ = **0.30** ± **0.04s**.

Beyond that, this study confirms ATT estimations of other, previously published results using pCASL (Guo et al., 2018) and PASL acquisitions (MacIntosh et al., 2010). Temporal differences of ATTs obtained from pCASL and PASL scans can be inferred from different gaps (∼Δ3 cm) between labeling and readout regions, method-related differences in the arterial flow profile, including dispersion effects (Zhang et al., 2021), and thereby also in the kinetic curve, which in turn affects the results of the kinetic modeling of perfusion parameters.

In alignment to the literature, we also confirmed in this study, that women tend to show shorter ATT (Liu et al., 2012) and higher CBF values (Parkes et al., 2004; Chen et al., 2011; Liu et al., 2012) for gray matter than men. With the help of one-way ANOVAs we found, that these gender differences on perfusion are more pronounced in pCASL scans from the Skyra (ATT with *p* = 0.0001, CBF with *p* = 0.001), compared to pCASL scans from the mMR Biograph (ATT with *p* = 0.027, CBF with *p* = 0.076) and compared to PASL scans (ATT with *p* = 0.0005, CBF with *p* = 0.016).

### Pseudo-Continuous ASL Reliability

**FIGURE 3:**
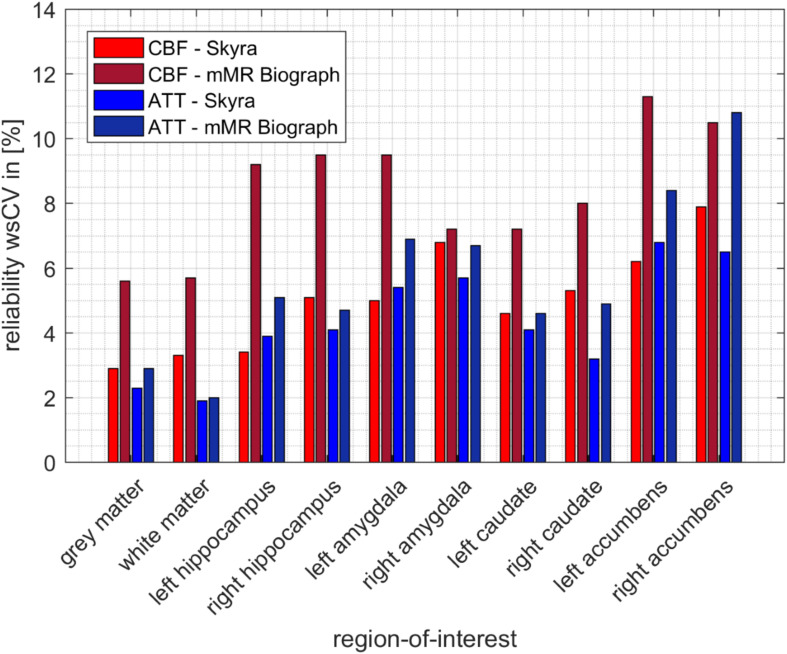
Visualized reliability of CBF (red) and ATT (blue) estimated from pCASL acquisitions (from Skyra and mMR Biograph) for several region-of-interests. Corresponding within-subject coefficients of variation (wsCV) values are listed in Table 2.

**FIGURE 4:**
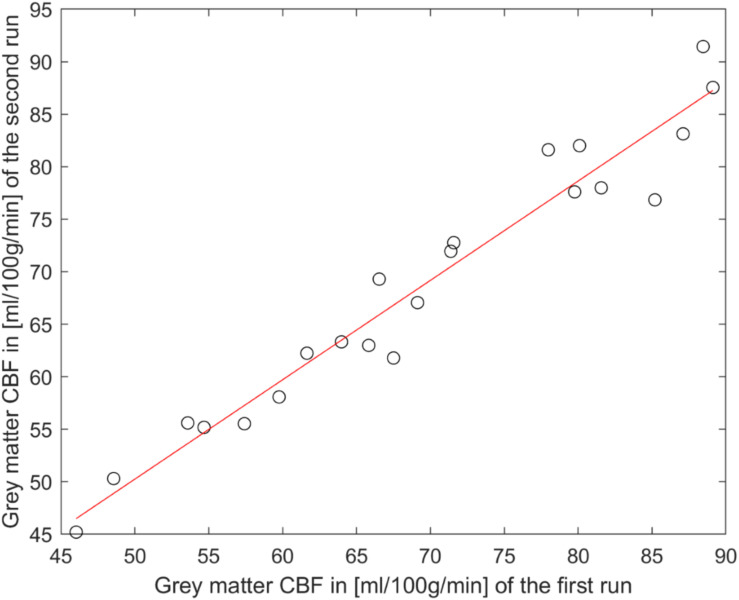
Excellent reliability of pCASL acquisitions at the Skyra: CBF values for the gray matter region-of-interest of the first and the second run of the same session is shown for every participant (circles). The straight red line represents a linear fit of these values.

The reliability of pCASL acquisitions was accessed by calculating within-subject coefficients of variation (wsCV) for mean CBF and ATT values for both runs (see Figure 3 and Table 2). Four participants measured at the Skyra were excluded from further reliability analysis (three had only one pCASL run and one participant showed un-physiological low CBF values in one run), which results in a total number of 22 participants for this wsCV analysis 23 participants participated in a second MRI session at an mMR Biograph, from which two participants were excluded because of incomplete data. Figure 4 shows a perfect agreement of estimated gray matter CBF values of both pCASL runs obtained at the Skyra for every participant.

**TABLE 2:** Reliability of the perfusion estimated from pseudo-continuous ASL (pCASL) acquisitions (from Skyra and mMR Biograph).

ROI	wsCV in [%]			
	
	CBF		ATT	
		
	Skyra	mMR Biograph	Skyra	mMR Biograph
Gray matter	2.9	5.6	2.3	2.9
White matter	3.3	5.7	1.9	2.0
Left hippocampus	3.4	9.2	3.9	5.1
Right hippocampus	5.1	9.5	4.1	4.7
Left amygdala	5.0	9.5	5.4	6.9
Right amygdala	6.8	7.2	5.7	6.7
Left caudate	4.6	7.2	4.1	4.6
Right caudate	5.3	8.0	3.2	4.9
Left accumbens	6.2	11.3	6.8	8.4
Right accumbens	7.9	10.5	6.5	10.8

*Low within-subject coefficients of variation (wsCV) for mean cerebral blood flow (CBF) or of arterial transit time (ATT) results indicate a high reliability. wsCV results are listed for several region-of-interests (ROI).*

Furthermore, the within-subject coefficient of variation of gray matter CBF perfectly confirms the reported values from Chen et al. (2011). They estimated an wsCV of 3.5% for pCASL acquisitions of 12 healthy young participants. Overall, Figure 3 and Table 2 show that CBF and ATT values obtained at the Skyra are more reliable than those from the mMR Biograph for all considered ROIs as indicated by lower wsCV values. This agrees with the observation of less participant motion during pCASL acquisitions at the Skyra (see above), although individual squared coefficient of variance (CV^2^) changes between scanners do not correlate with translation changes in y-direction (*p* = 0.084 for CBF and *p* = 0.914 for ATT with gender as covariate).

### Pseudo-Continuous ASL Reproducibility

**FIGURE 5:**
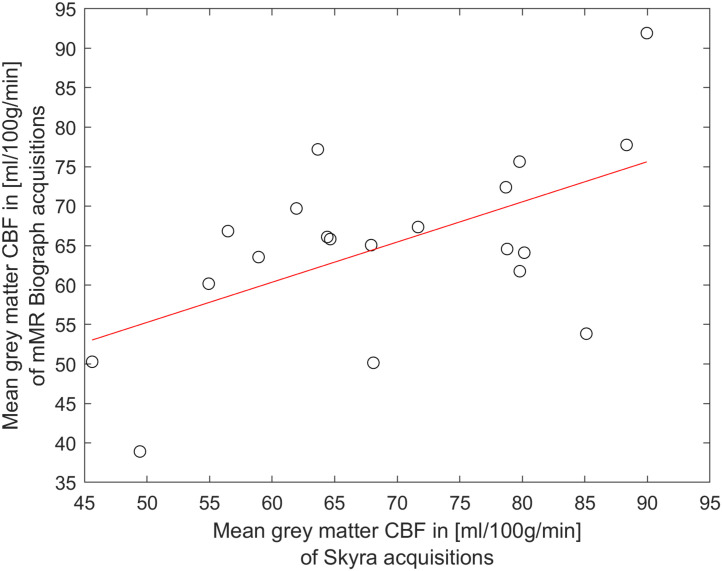
Substantial reproducibility of pCASL acquisitions: The mean CBF of both runs for the gray matter region-of-interest from Skyra acquisitions is compared to those from mMR Biograph acquisitions. A circle represents one participant. The straight red line is the linear fit of these values.

The mean of both pCASL runs from the Skyra was calculated for CBF and ATT, respectively, and then compared to the mean of both pCASL runs from the mMR Biograph (see Figure 5). The pCASL reproducibility was evaluated by means of wsCV and ICC analysis (results are shown in Table 3). Data in all ROIs passed Shapiro–Wilk tests for normality (*p* > 0.05), except ATT data for left and right caudate and right accumbens (corresponding results are only listed for completeness). According to Landis and Koch (1977) ICC results between 0.81 and 1.00 were assigned to be “near perfect,” ICC between 0.61 and 0.8 were assigned to be “substantial” and ICC results between 0.41 and 0.6 were assigned to be “moderate”. Table 3 shows an overall good reproducibility of CBF and ATT measures. However, in all cases ATT values were more replicable than CBF indicated by far lower wsCV values. Additionally, the good reproducibility was also confirmed by high ICC values denoting a substantial agreement of CBF and ATT values between both sessions and systems, respectively.

**TABLE 3:** Reproducibility of perfusion measures from pseudo-continuous ASL (pCASL) acquisitions.

ROI	wsCV in [%]		ICC (95% confidence intervals)	
		
	CBF	ATT	CBF	ATT
Gray matter	12.7	5.5	0.704 (0.282 to 0.881)	0.754 (0.190 to 0.913)
White matter	13.2	3.6	0.758 (0.396 to 0.904)	0.713 (0.198 to 0.891)
Left hippocampus	14.8	8.4	0.647 (0.146 to 0.858)	0.607 (0.032 to 0.843)
Right hippocampus	13.9	10.1	0.612 (0.077 to 0.843)	0.547 (−0.174 to 0.828)
Left amygdala	17.0	5.8	0.374 (−0.351 to 0.734)	0.775 (0.440 to 0.910)
Right amygdala	14.8	8.0	0.369 (−0.510 to 0.744)	0.655 (0.172 to 0.860)
Left caudate	14.1	5.6	0.670 (0.148 to 0.870)	0.875 (0.687 to 0.950)
Right caudate	12.8	6.0	0.737 (0.326 to 0.896)	0.865 (0.608 to 0.949)
Left accumbens	16.6	8.3	0.663 (0.126 to 0.868)	0.806 (0.508 to 0.924)
Right accumbens	14.2	8.6	0.612 (0.011 to 0.847)	0.821 (0.554 to 0.929)

*Mean cerebral blood flow (CBF) and arterial-transit-time (ATT) values from a Skyra MRI system were compared to mean CBF and ATT values from an mMR Biograph system. Low within-subject-coefficients of variation (wsCV) as well as high intra-class correlation (ICC) coefficients indicate a high reproducibility. wsCV and ICC were calculated for several region-of-interests (ROI). ATTs for the caudate and right accumbens did not pass Shapiro–Wilk tests for normality and corresponding ICC results are just listed for completeness.*

All in all, the obtained wsCV for gray matter agree with those recently published by Cohen et al. (2020) although they used a shorter inter-session interval and all sessions were conducted on the same system. The ICC coefficient of gray matter also matches the one published by Cohen et al. (2020). Nevertheless, we see a difference in the calculated ICCs of gray matter for the ATT: We found a similar or even higher ICC of gray matter for the ATT (0.754) compared to CBF (0.704), whereas Cohen et al. (2020) reported a significantly lower ICC for the ATT (0.49). Another, only recently published study on elderly participants reported also a comparable ICC coefficient of 0.77 for white matter CBF values obtained in two sessions around six weeks apart on the same MRI system (Jann et al., 2021).

### Comparison of pCASL and PASL Results

Mean CBF values from both pCASL runs from the Skyra were compared to PASL perfusion results via ICC calculations (Table 4). Participants with only one pCASL run (see above) were included in this analysis, too, which results in a total number of 23 participants. Data in all ROIs passed Shapiro–Wilk tests for normality (*p* > 0.05).

**TABLE 4:** Comparison of estimated cerebral blood flow (CBF) values obtained with a pseudo-continuous ASL (pCASL) sequence to those obtained with a pulsed ASL (PASL) sequence at the Skyra MRI system.

ROI	ICC (95% confidence intervals)
Gray matter	0.830 (0.589 to 0.930)
White matter	0.470 (−0.093 to 0.827)
Left Hippocampus	0.633 (−0.225 to 0.890)
Right Hippocampus	0.514 (−0.241 to 0.828)
Left Amygdala	0.584 (0.044 to 0.824)
Right Amygdala	0.580 (−0.127 to 0.839)
Left Caudate	0.591 (−0.154 to 0.848)
Right Caudate	0.758 (0.419 to 0.899)
Left Accumbens	0.509 (−0.212 to 0.808)
Right Accumbens	0.802 (0.532 to 0.917)

*Corresponding intra-class correlation (ICC) coefficients are listed for several region-of-interests (ROI).*

In all ROIs the agreement of CBF values was at least moderate. In gray matter the consistency between mean CBF values of different ASL labeling approaches was even near perfect.

### Cerebral Blood Flow and Arterial Transit Time Correlation

The influence of ATT estimations on CBF quantification results was evaluated with the help of Pearson’s correlation coefficients. As the cerebral perfusion is dependent on the participant’s age and gender (Shin et al., 2007), we also included both parameters as covariates in the analysis. The results for various ROIs are listed in Table 5.

**TABLE 5:** Pearson’s correlation coefficients of cerebral blood flow (CBF) and arterial transit time (ATT) with (“wC”) and without (“woC”) controlling for the covariates age and gender for pulsed ASL (PASL) and pseudo-continuous ASL (pCASL) scans at the magnetic resonance imaging (MRI) system Skyra and mMR Biograph.

ROI	Pearson’s correlation coefficient of CBF with ATT									
	
	Skyra						mMR Biograph			
		
	PASL		pCASL run1		pCASL run2		pCASL run1		pCASL run2	
					
	woC	wC	woC	wC	woC	wC	woC	wC	woC	wC
Whole brain			−0.53		−0.66		−0.64	−0.52	−0.60	
Gray matter	−0,49		−0.61		−0.73		−0.67	−0.55	−0.64	−0.45
White matter	−0.68		−0.81	−0.65	−0.89	−0.68	−0.83	−0.72	−0.75	−0.59
Left hippocampal	−0.43		−0.50		−0.55		−0.54		−0.47	
Right hippocampal	−0.53		−0.57		−0.61		−0.69	−0.57	−0.57	−0.46
Left amygdala	−0.52		−0.67	−0.45	−0.57				−0.56	
Right amygdala			−0.48		−0.56		−0.71	−0.61	−0.43	
Left caudate			−0.68	−0.51	−0.70	−0.45	−0.72	−0.62	−0.72	−0.58
Right caudate			−0.61	−0.84	−0.79	−0.47	−0.69	−0.54	−0.67	
Left accumbens			−0.57		−0.57	−0.46	−0.62	−0.52	−0.62	−0.61
Right accumbens			−0.40		−0.61				−0.68	−0.58

*Correlation coefficients are calculated for several region-of-interests (ROI) and only significant results (*p* < 0.05) are listed. Levels of significance are omitted for clarity.*

All in all, we observed a strong negative correlation of CBF and ATT, especially for gray and white matter ROIs, which is more pronounced in pCASL than in PASL acquisitions. This confirms the observation only recently published by Cohen et al. (2020). The incorporation of age and gender dependencies led to a slight reduction of the relationship between ATT and CBF.

## Discussion

We investigated the cerebral perfusion obtained with a 7-block Hadamard encoded 3D pCASL sequence on a group of healthy elderly at two different 3T Siemens MR systems (Skyra, mMR Biograph). All in all, we found an excellent intra-session reliability of CBF and ATT, and a considerable agreement of perfusion parameters acquired in separate sessions and on different MR systems. Although the mean ATT for gray and white matter, as well as for left and right hippocampus, was slightly larger in Skyra acquisitions, an equivalent analysis for the mean CBF showed only a significant difference for the left amygdala (*p* = 0.019). It is possible that the labeling planes for the pCASL scans at the Skyra might have been positioned inferior to those at the mMR Biograph and longer ATTs may therefore result from a longer distance the bolus had to pass. Encouragingly, these longer ATTs had almost no effect on the CBF quantification results, which is important for example in multi-center studies where different operators conduct the scans.

Nevertheless, comparing the pCASL reliability of Skyra and mMR Biograph, we found that CBF and ATT estimations were even more reliable if acquired at the Skyra (smaller wsCV). We additionally observed less participant motion in Skyra scans, which might have been an effect of different methods in participant positioning and fixation or of individual differences how radiographers instructed and motivated participating volunteers. Although participants’ motion during MRI acquisitions has an effect on the signal variance and therefore also on reliability values, we could not show a clear relationship between wsCV and these motion parameters, indicating that the contribution of (other) physiological sources to the total signal variance dominated.

Besides the fact, that participants’ motion differed significantly between Skyra and mMR Biograph acquisitions, the overall reproducibility of pCASL scans between both systems was of a substantial agreement, which encourages the use of this pCASL sequence in multi-center perfusion studies. Moreover, obtained reproducibility measures were comparable to values previously published by Cohen et al. (2020) and Jann et al. (2021). Interestingly, reproducibility values obtained in these studies matches those of our study although we used a longer inter-session interval of 78 ± 17.6 days (in Cohen’s study up to 45 days, in Jann’s study around 47 days) and additionally conducted both sessions on two different MRI systems. In our case, the total signal variance included therefore not only physiological but also technical contributions. One of the limitations of our study is, that we neither controlled for participants’ caffeine uptake in the time before the ASL measurements took place, nor minimized diurnal fluctuations by choosing the same time of the day for the two sessions. It is well known that caffeine affects cerebral perfusion as published by Mutsaerts et al. (2015) and that diurnal fluctuations might also have an influence as mentioned by Ssali et al. (2016).

Altogether, ATT appears to be less sensitive to physiological alterations than CBF. In our study we could confirm the results shown in Cohen et al. (2020), that ATT was more reliable than CBF. This further approves, that the ATT should be accounted for in perfusion studies, especially in those investigating aging (Juttukonda et al., 2021) and elderly (Dai et al., 2017) or patients suffering from neurodegenerative diseases (Suo et al., 2019).

There are two reasons to estimate ATT: It is a valuable hemodynamic parameter as noted above, and a correct ATT is sensible for the CBF quantification. We showed a strong negative correlation of ATT and CBF (Table 5): Longer ATTs were associated with a decrease in CBF. The central volume principle (Meier and Zierler, 1954) provides the theoretical basis for this relation. Furthermore, this ATT-CBF correlation is possibly also related to a greater vessel tortuosity and a simultaneous CBF decrease in old age. Wenn and Newman (1990) observed a greater vessel tortuosity associated with a reduced stiffness of vessel walls. Especially for participants with an age of 40 or higher this tortuosity increases enormously (Wenn and Newman, 1990). An age related increase of the vessel tortuosity would be reflected by a longer stretch of vessel way for the same distance, and therefore by longer ATTs. Together with an additional observed decrease in CBF in elderly (Lu et al., 2011) this may be a factor for the negative correlation of ATT and CBF. The influence of age and gender to the correlation results of ATT and CBF is also shown in Table 5 as Pearson’s correlation coefficients are decreased if age and gender are incorporated into the model.

If the labeled blood bolus had been sampled incompletely due to inadequate choice of PLDs, longer ATTs would also have inherently led to a CBF underestimation although ATT variations were accounted for in the CBF modeling. To confirm a sufficient bolus sampling we plotted the mean gray matter intensities of the perfusion difference images for every inversion time/PLD for all participants. Nevertheless, ATT estimates might be additionally slightly biased by the choice of the initial ATT value within the perfusion modeling. Future studies should investigate the relation of ATT prior values to perfusion results.

All in all, the obtained negative correlation of ATT and CBF was more pronounced in pCASL than in PASL scans. Paling et al. (2014) found an inverse relation of ATT and CBF in patients suffering from MS measured with multi-TI PASL. Furthermore, Mutsaerts et al. (2017) could show this ATT-CBF correlation in a study about elderly with hypertension, whereas this relation could also be referred for healthy, young participants (Donahue et al., 2014).

In summary, we investigated a 7-block Hadamard encoded pCASL sequence on a group of healthy elderly on two different 3T Siemens MRI systems (Skyra and mMR Biograph). An excellent test-retest reliability of the cerebral perfusion was shown for both systems. We found ATT to be the more reliable perfusion parameter compared to CBF. Particularly in studies investigating the relation of perfusion and neurodegenerative disease, ATT could give valuable information. The substantial agreement of the perfusion results (CBF and ATT) from both MRI systems confirms the usability of this innovative sequence for multi-center perfusion studies.

## Data Availability Statement

The original contributions presented in the study are included in the article/supplementary material, further inquiries can be directed to the corresponding author.

## Ethics Statement

The studies involving human participants were reviewed and approved by Ethics Committee of the Otto von Guericke University Magdeburg, Germany. The patients/participants provided their written informed consent to participate in this study.

## Author Contributions

KN analyzed the data and wrote the manuscript. MS generated the used pCASL sequence including the MRI protocol and analyzed the data. TS generated the used pCASL sequence including the MRI protocol. MG generated the used PASL sequence. All authors discussed the results and proof-read this article.

## Conflict of Interest

MG receives royalties from Siemens Healthineers for technology using ASL. The remaining authors declare that the research was conducted in the absence of any commercial or financial relationships that could be construed as a potential conflict of interest.

## Publisher’s Note

All claims expressed in this article are solely those of the authors and do not necessarily represent those of their affiliated organizations, or those of the publisher, the editors and the reviewers. Any product that may be evaluated in this article, or claim that may be made by its manufacturer, is not guaranteed or endorsed by the publisher.
